# Less frequent skin ulcers among patients with Werner syndrome treated with pioglitazone: findings from the Japanese Werner Syndrome Registry

**DOI:** 10.18632/aging.206161

**Published:** 2024-12-02

**Authors:** Kazuto Aono, Masaya Koshizaka, Mayumi Shoji, Hiyori Kaneko, Yukari Maeda, Hisaya Kato, Yoshiro Maezawa, Makoto Miyabayashi, Mai Ishikawa, Akiko Sekiguchi, Sei-Ichiro Motegi, Shinji Tsukamoto, Akira Taniguchi, Yukiko Shoda, Toru Yoshimura, Junji Kawashima, Kayo Yoshinaga, Hironori Nakagami, Yoichi Takami, Ken Sugimoto, Kunihiko Hashimoto, Naoki Okubo, Takashi Yoshida, Masato Ohara, Asako Kogure, Daisuke Suzuki, Masafumi Kuzuya, Kazuhisa Watanabe, Minoru Takemoto, Junko Oshima, Koutaro Yokote

**Affiliations:** 1 Department of Endocrinology, Hematology and Gerontology, Chiba University Graduate School of Medicine, Chiba, Japan; 2 Center for Preventive Medical Sciences, Chiba University, Chiba, Japan; 3 Clinical Research Center, Chiba University Hospital, Chiba, Japan; 4 Department of Dermatology, Gunma University Graduate School of Medicine, Maebashi, Japan; 5 Department of Orthopaedic Surgery, Nara Medical University, Nara, Japan; 6 Department of Dermatology, Sumitomo Hospital, Osaka, Japan; 7 Diabetes and Endocrinology, Saga-Ken Medical Centre Koseikan, Saga, Japan; 8 Department of Metabolic Medicine, Faculty of Life Sciences, Kumamoto University, Kumamoto, Japan; 9 Department of Health Development and Medicine, Osaka University Graduate School of Medicine, Osaka, Japan; 10 Department of Geriatric and General Medicine, Osaka University Graduate School of Medicine, Osaka, Japan; 11 General Geriatric Medicine, Kawasaki Medical School, Okayama, Japan; 12 Department of Endocrinology and Metabolic Medicine, Nippon Life Hospital, Osaka, Japan; 13 Department of Orthopaedics, Graduate School of Medical Science, Kyoto Prefectural University of Medicine, Kyoto, Japan; 14 Department of Orthopaedic Surgery, North Medical Center, Kyoto Prefectural University of Medicine, Kyoto, Japan; 15 Department of Dermatology, Showa General Hospital, Tokyo, Japan; 16 Geriatrics and General Internal Medicine, Meitetsu Hospital, Nagoya, Japan; 17 Department of Community Healthcare and Geriatrics, Nagoya University Graduate School of Medicine, Nagoya, Japan; 18 Department of Medicine, Division of Diabetes, Metabolism and Endocrinology, International University of Health and Welfare, Narita, Japan; 19 Department of Laboratory Medicine and Pathology, University of Washington, Seattle, WA 98195, USA

**Keywords:** Werner syndrome, skin ulcer, metformin, pioglitazon, progeroid syndrome

## Abstract

Background and Aim: Werner syndrome (WS) is an autosomal recessive, adult-onset, progeroid syndrome caused by *WRN* mutations. As refractory skin ulcers significantly affect the quality of life of patients with WS, this study identified ulcer risk factors and assessed prevention methods.

Methods: We analyzed the data of 51 patients with WS enrolled in the Japanese Werner Syndrome Registry between 2016 and 2022. A cross-sectional analysis was performed to determine the association with skin ulcers at baseline. Statistical analyses were conducted, including Welch’s and Pearson’s chi-square tests. Age was adjusted using a logistic regression model.

Results: The mean patient age was 48.8±7.6 years, and 66.7% of patients presented with skin ulcers. Univariate analysis showed that patients with skin ulcers were older than those without ulcers. Systolic blood pressure (SBP) was higher in patients with skin ulcers. Patients without skin ulcers received metformin and pioglitazone treatment significantly more often than those with ulcers. Logistic regression analysis adjusted for age showed that higher SBP remained a significant risk factor for skin ulcers. Patients administered pioglitazone had lower ulcer morbidity.

Conclusions: Age and SBP are risk factors for skin ulcers in patients with WS. Moreover, pioglitazone treatment may prevent skin ulcers.

## INTRODUCTION

Werner syndrome (WS) is an autosomal recessive adult-onset progeroid syndrome caused by *WRN* mutations. Patients with WS present various signs of aging, including graying, hair loss, and bilateral juvenile cataracts, which appear during the second decade of life. Previously, the average expectancy was in the 40s owing to myocardial infarction and malignancy. However, recently, the average reported lifespan has extended to 59 years owing to therapeutic advances that have substantially reduced the incidence of arteriosclerotic diseases [1]. However, refractory skin ulcers, a characteristic symptom of WS, occur in 67.5% of patients, causing severe pain and requiring limb amputation in 15% of patients [2, 3]. There is no fundamental treatment for refractory skin ulcers. Therefore, patients with WS and refractory skin ulcers have a significantly diminished quality of life owing to pain and activity limitations [4]. Given the expanding life expectancy of patients with WS, prevention and treatment of refractory skin ulcers are crucial for enhancing patients’ overall quality of life. Regarding patient and public involvement, we regularly interact with patients with WS registered in the Japanese Werner Syndrome Patients and Family Association, who have increasingly requested treatment for refractory ulcers. However, the risk factors for developing refractory skin ulcers in patients with WS remain unknown. Therefore, this cross-sectional study used clinical data from patients with WS in the Japanese Werner Syndrome Registry to elucidate the risk factors for skin ulcers by analyzing background factors in patients with and without skin ulcers.

## RESULTS

Among 51 patients enrolled from the Japanese Werner Syndrome Registry, the mean age was 48.8±7.6 years, 52.9% were male individuals, and 66.7% presented with skin ulcers. The results of the univariate analysis of patient characteristics with and without skin ulcers are shown in Table 1. Patients with skin ulcers were significantly older than those without ulcers (50.6±6.8 years vs. 45.1±8.0 years, *P*=0.02). Lipid levels, including low-density lipoprotein cholesterol, high-density lipoprotein cholesterol, and triglyceride, did not differ between the groups. Regarding renal function, blood urea nitrogen levels were significantly higher (17.6±1.3 mg/dL vs. 13.7±1.9 mg/dL, *P*=0.03). Systolic blood pressure (SBP) was also significantly higher in patients with skin ulcers (129.4±19.6 mmHg vs. 111.3±9.6 mmHg, *P*<0.01). Although hypertension tended to be more prevalent in patients with skin ulcers, no significant differences in morbidity due to diabetes, dyslipidemia, or peripheral artery disease (PAD) were observed. Patients without skin ulcers received treatment with metformin (43.8% vs. 15.6%, *P*=0.03) and pioglitazone (50.0% vs. 21.9%, *P*=0.04) compared with those with ulcers.

**Table 1:** Clinical characteristics of patients with and without skin ulcers.

	**N**	**Skin ulcers** **(mean±standard deviation or %)**	**N**	**Without skin ulcers (mean±standard deviation or %)**	***P*-value**
Age (years)	34	50.6±6.8	17	45.1±8.0	0.02
Male (%)	34	41.2	17	58.8	0.23
Body mass index (kg/m^2^)	34	18.2±3.1	17	18.4±3.0	0.82
Waist circumference (cm)	18	78.2±11.7	12	75.6±10.3	0.51
HDL-C (mg/dL)	29	56.9±21.3	17	58.2±20.2	0.84
LDL-C (mg/dL)	26	122.3±26.1	13	112.6±27.7	0.25
Triglyceride (mg/dL)	32	151±78.7	17	181±121.9	0.36
Albumin (mg/dL)	32	4.2±0.8	16	4.5±0.5	0.10
BUN (mg/dL)	33	17.6±1.3	15	13.7±1.9	0.03
Creatinine (mg/dL)	34	0.86±1.0	16	0.59±0.2	0.13
Fasting plasma glucose (mg/dL)	11	122±27.1	10	122.7±31.8	0.48
HbA1c (%)	29	6.3±1.0	17	6.6±1.6	0.54
SBP (mmHg)	30	129.4±19.6	15	111.3±9.6	<0.01
DBP (mmHg)	30	71±12.6	15	64.4±11.7	0.09
Hypertension (%)	32	46.9	16	25	0.14
Diabetes (%)	34	67.6	17	70.6	0.83
Metformin user (%)	32	15.6	16	43.8	0.03
Pioglitazone user (%)	32	21.9	16	50	0.04
Dyslipidemia (%)	34	67.8	17	64.7	0.83
Statin user (%)	32	43.8	16	37.5	0.67
Foot amputation (%)	34	17.7	17	5.9	0.25
PAD (%)	34	14.7	17	5.9	0.35

BUN, blood urea nitrogen; DBP, diastolic blood pressure; HbA1c, glycated hemoglobin; HDL-C, high-density lipoprotein cholesterol; LDL-C, low-density lipoprotein cholesterol; PAD, peripheral artery disease; SBP, systolic blood pressure.

Given the age-associated increase in ulcers, Table 2 presents the odds ratios (ORs) and 95% confidence intervals (CIs) calculated from the logistic regression analyses of skin ulcers adjusted for age. Even after adjusting for age, higher SBP remained a significant risk factor for skin ulcers (OR 1.08, 95% CI: 1.02–1.16, *P*=0.01), with diastolic blood pressure also showing tendency of increased risk for skin ulcers (OR 1.08, 95% CI: 1.00–1.16, *P*=0.06). Patients treated with pioglitazone still had a significantly lower risk of foot ulcers (OR 0.13, 95% CI 0.02–0.72, *P*=0.02), and those treated with metformin showed a trend toward a lower risk.

**Table 2:** Odds ratios and 95% confidence intervals for skin ulcers adjusted for age.

	**OR (95% CI)**	***P*-value**
LDL-C	1.03 (1.00–1.06)	0.12
Albumin	0.81 (0.27–2.44)	0.71
BUN	1.12 (0.96–1.32)	0.14
Creatinine	12.5 (0.37–424)	0.16
SBP	1.08 (1.02–1.16)	0.01
DBP	1.08 (1.00–1.16)	0.06
Hypertension	1.50 (0.34–6.55)	0.59
Metformin user	0.31 (0.07–1.30)	0.11
Pioglitazone user	0.13 (0.02–0.72)	0.02

CI, confidence interval; BUN, blood urea nitrogen; DBP, diastolic blood pressure; LDL-C, low-density lipoprotein cholesterol; OR, odds ratio; SBP, systolic blood pressure.

## DISCUSSION

**Figure 1:**
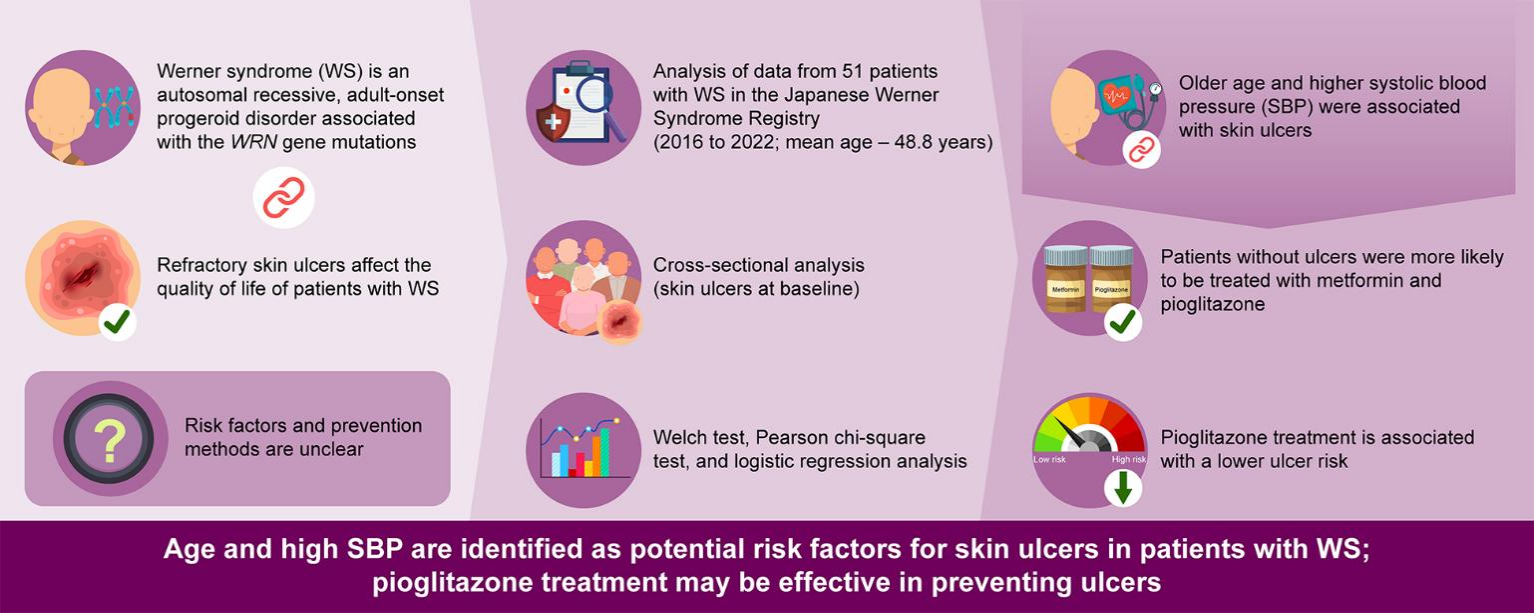
Graphical abstract of this study.

This study retrospectively investigated the background factors associated with skin ulcers in patients with WS. Patients with skin ulcers were significantly older and had higher SBP, while those without skin ulcers were significantly more likely to receive treatment with pioglitazone and metformin. Even after adjusting for age, treatment with pioglitazone remained significantly associated with a reduced risk of ulcers. These data suggest that pioglitazone treatment prevents the development of skin ulcers (Figure 1).

The mechanism of the refractory skin ulcers characteristic of WS may involve multiple factors, including subcutaneous fat atrophy and impaired blood flow associated with arteriosclerotic disease. Despite having a low body mass index, patients with WS often have visceral fat accumulation, and subcutaneous fat atrophy is observed in the extremities compared with the trunk. *WRN* deficiency promotes adipocyte maturation, possibly by altering chromatin accessibility [5]. Kato et al. reported decreased proliferative potential and adipogenic differentiation capacity in foot-derived fibroblasts compared with trunk-derived fibroblasts in patients with WS, which may lead to refractory skin ulcers. In addition, the expression of peroxisome proliferator-activated receptor gamma (PPARγ), which governs adipose synthesis, was decreased in foot-derived fibroblasts compared with trunk-derived fibroblasts, suggesting an association with fat atrophy [6]. Moreover, the stromal vascular fraction (SVF) derived from the subcutaneous fat of patients with WS showed suppressed adipogenesis and increased expression of the senescence-associated secretory phenotype compared with that derived from the tissues of healthy individuals [7]. Therefore, subcutaneous fat atrophy observed in patients with WS may contribute significantly to the development of skin ulcers.

The risk factors for skin ulcers in patients with WS have not been well elucidated, although diabetic foot ulcers, a major cause of skin ulcers, have been investigated. The risk factors for diabetic foot ulcers include smoking, male sex, older age, diabetes duration, poor glycemic control, hypertension, PAD, and sarcopenia [8–11].

The present study identified older age and hypertension as risk factors for foot ulcers in patients with WS. As far as we know, no other studies have reported the effects of age and blood pressure on skin ulcers in patients with WS. However, the following mechanisms explain the association of foot ulcers with respect to age: impaired blood circulation is a common cause of ulcerations. Age-related decrease in blood vessel function can result in poor circulation in the foot, leading to an inadequate supply of oxygen and nutrients to tissues. Moreover, the skin loses elasticity and becomes more prone to dryness with age, thus increasing the risk for minor injuries to progress to ulcers. Additionally, high blood pressure affects an increased risk of leg ulcers in patients with diabetes, with significant negative effects on healing time [12]. This effect may occur because persistently high blood pressure can increase the strain on blood vessels, potentially leading to vascular damage and atherosclerosis, which may compromise blood flow to the lower limbs and delay or exacerbate ulcer healing. High blood pressure may also influence immune response, which can affect ulcer healing. Therefore, foot ulcers in patients with WS and higher SBP require attention.

While the incidence of PAD did not differ significantly, it tended to be more prevalent in patients with skin ulcers. Significant differences were observed as the sample size increased. The lack of significant difference in diabetes mellitus may be explained by good glycemic control in both groups. However, no sex-related differences were observed between groups. Phenotypic and sex-based differences in WS have attracted considerable attention in recent years [13]. Further research is needed to determine whether the sex differences reported in healthy individuals are also present in WS.

Skin ulcers in WS are refractory to medical therapy. These patients often require surgical treatment, including amputation [14]. The current study findings showed that patients without skin ulcers more often received metformin or pioglitazone treatment.

Pioglitazone is suitable for treatment for patients with WS who have insulin-resistant diabetes in the point of ameliorating insulin resistance [15, 16]. Previous reports have found metformin and pioglitazone to be useful in treating wounds. For instance, Zhao et al. reported that the systemic administration of metformin increased angiogenesis at the wound site and promoted wound healing in young mice. Interestingly, local administration of metformin also increased epidermal thickness and collagen deposition in the skin in old mice, indicating that metformin strongly promotes wound healing [17]. One possible mechanism is that activation of the adenosine monophosphate-activated protein kinase pathway by metformin enhances anti-inflammatory M2 macrophages polarization by suppressing the activation of nucleotide-binding oligomerization domain-like receptor family pyrin domain-containing (NLRP) inflammasomes [18]. Clinical trials of topical metformin and metformin hydrochloride hydrogels have also demonstrated their usefulness in treating trauma and skin ulcers [19].

Several reports have also shown that topical pioglitazone promotes wound healing in the skin ulcers of diabetic mice [20–22]. The NLRP3 inflammasome-interleukin-1β (IL-1β) pathway is active in the diabetic wound environment, and IL-1β downregulates macrophage PPARγ activity; this reduced PPARγ activity results in prolonged inflammation and delayed wound healing [22]. Pioglitazone is a PPAR agonist; thus, PPARγ activation has an anti-inflammatory effect by inducing the differentiation of monocytes into M2 macrophages [23].

To our knowledge, no studies have evaluated the efficacy of metformin or pioglitazone in the treatment of WS skin ulcers. However, the SVF extracted from the subcutaneous fat of patients with WS showed significantly increased IL-1β expression, suggesting that the wound environment in WS is also prone to prolonged inflammation due to PPARγ suppression [7]. Therefore, pioglitazone may be effective in the preventing and treating of skin ulcers in patients with WS. A typical side effect of oral pioglitazone is decreased bone density. As patients with WS often have significant osteoporosis, especially in the femur, treatment with pioglitazone may worsen this condition [24]; however, topical administration may reduce these adverse effects. Nanostructured hybrid materials loaded with pioglitazone are also being developed for clinical use and may be useful as wound dressings for ulcer treatment [25].

The present study has some limitations. First, the sample size included in the analysis was limited because of the rarity of WS. Therefore, these results require careful interpretation. However, this registry is a large WS database. Second, we conducted a cross-sectional analysis of registry data with medication only at the time of registration and without a past medication history. This makes it difficult to establish a causal relationship between the development of skin ulcers and risk factors or previous medications. However, further prospective studies are required to confirm this association. Finally, this study used only questionnaire information, which lacks detailed data such as smoking history, duration of diabetes, or ulcer severity; therefore, performing further analysis or excluding the influence of unknown confounding factors is complicated.

However, the results of this study indicate that pioglitazone might be useful in treating refractory skin ulcers, a typical condition that reduces the quality of life of patients with WS. Future basic research should investigate whether pioglitazone treatment has beneficial effects on WS-derived fibroblasts, adipose tissue, and macrophages. In a future prospective interventional study, we hope to confirm the potential usefulness of topical pioglitazone for the treatment of skin ulcers in WS.

In conclusion, patients with WS, age and SBP are risk factors for skin ulcers. Moreover, pioglitazone may be effective in treating skin ulcers. Additional prospective studies are required to investigate the causal relationship between the development of skin ulcers and risk factors or previous medications. Future studies are also needed to demonstrate the potential benefits of pioglitazone use on WS-derived fibroblasts, adipose tissue, and macrophages and to confirm the usefulness of topical pioglitazone to treat skin ulcers in patients with WS.

## MATERIALS AND METHODS

The Japanese Werner Syndrome Registry, conducted in collaboration with 13 Japanese hospitals, is currently underway to investigate WS, enroll participants for clinical trials, and share information to enrolled patients and clinicians. This critical inclusion criterion comprises patients meeting two conditions: 1) confirmation of WS by the diagnostic criteria [26] and 2) provision of informed written consent before participating in the registry. The diagnosis of WS was confirmed by physicians, dependeding on all cardinal signs are present or a gene mutation in addition to at least three cardinal signs. The present study had no exclusion criteria.

The current investigation analyzed data from 51 participants enrolled in the Japanese Werner Syndrome Registry from 2016 to 2022. The years of patient registration are as follows: 9 patients in 2016, 6 in 2017, 17 in 2018, 6 in 2019, 4 in 2020, 6 in 2021, and 3 in 2022. Data from the initial survey were analyzed for associations with skin ulcers. The data included patient background details such as age at registration, sex, body mass index, and abdominal circumference; clinical symptoms; comorbidities, including hypertension, diabetes mellitus, dyslipidemia, foot amputation, and PAD; blood examination results, including renal function, glucose metabolism, and lipid profiles; and treatment details. The comorbidities were diagnosed by physicians based on each diagnostic criterion. Renal function was evaluated by blood urea nitrogen and creatinine. Glucose metabolism was evaluated by fasting plasma glucose and glycated hemoglobin levels. Regarding lipid profiles, triglyceride, high-density lipoprotein cholesterol, and low-density lipoprotein cholesterol were measured. Blood examinations were performed as possible as in fasting condition. Blood pressures were assessed while the patients were seated.

Comparative analyses of background factors were conducted between groups with and without skin ulcers. Statistical analysis using JMP Pro version 15 (SAS Institute, Cary, NC, USA) involved the Welch test for continuous variables and the Pearson chi-square test for binary variables. Age adjustment was conducted using a logistic regression model, and ORs and 95% CIs were estimated using a Wald test. All statistical tests were two-tailed, and *P*<0.05 was considered statistically significant.

### Data availability statement

The datasets generated during and/or analyzed during the current study are available as collaboration studies with Japanese Werner Syndrome Registry study group on reasonable request.
